# Electric dipole induced bulk ferromagnetism in dimer Mott molecular compounds

**DOI:** 10.1038/s41598-020-79262-6

**Published:** 2021-01-14

**Authors:** Ryo Yoshimoto, Satoshi Yamashita, Hiroki Akutsu, Yasuhiro Nakazawa, Tetsuro Kusamoto, Yugo Oshima, Takehito Nakano, Hiroshi M. Yamamoto, Reizo Kato

**Affiliations:** 1 Department of Chemistry, Graduate School of Science, Osaka University, Toyonaka, Osaka 560-0043 Japan; 2 Department of Life and Coordination-Complex Molecular Science, Institute for Molecular Science, Okazaki, Aichi 444-8787 Japan; 3 RIKEN, Cluster for Pioneering Research (CPR), Wako, Saitama 351-0198 Japan; 4 Institute of Quantum Beam Science, Graduate School of Science and Engineering, Ibaraki University, Mito, Ibaraki 310-8512 Japan; 5 Research Center of Integrative Molecular Systems (CIMoS), Institute for Molecular Science, Okazaki, Aichi 444-8585 Japan

**Keywords:** Ferromagnetism, Magnetic properties and materials

## Abstract

Magnetic properties of Mott–Hubbard systems are generally dominated by strong antiferromagnetic interactions produced by the Coulomb repulsion of electrons. Although theoretical possibility of a ferromagnetic ground state has been suggested by Nagaoka and Penn as single-hole doping in a Mott insulator, experimental realization has not been reported more than half century. We report the first experimental possibility of such ferromagnetism in a molecular Mott insulator with an extremely light and homogeneous hole-doping in π-electron layers induced by net polarization of counterions. A series of Ni(dmit)_2_ anion radical salts with organic cations, where dmit is 1,3-dithiole-2-thione-4,5-dithiolate can form bi-layer structure with polarized cation layers. Heat capacity, magnetization, and ESR measurements substantiated the formation of a bulk ferromagnetic state around 1.0 K with quite soft magnetization versus magnetic field (*M*–*H*) characteristics in (Et-4BrT)[Ni(dmit)_2_]_2_ where Et-4BrT is ethyl-4-bromothiazolium. The variation of the magnitude of net polarizations by using the difference of counter cations revealed the systematic change of the ground state from antiferromagnetic one to ferromagnetic one. We also report emergence of metallic states through further doping and applying external pressures for this doping induced ferromagnetic state. The realization of ferromagnetic state in Nagaoka–Penn mechanism can paves a way for designing new molecules-based ferromagnets in future.

## Introduction

The role of ferromagnets becomes more and more important as the electronics/spintronics and motorization technologies are advanced. Although microscopic mechanisms for ferromagnetic interaction are limited to several types so far^1–6^, theoretical proposals for different types of ferromagnets have been made analytically in Hubbard Hamiltonian. Nagaoka proposed ferromagnetism in a quite lightly doped Mott insulator in 1966, where only one hole is added to a Mott-insulator^7^. Similar result was reported by Penn almost the same time^8^. Tasaki proposed another type of ferromagnetism in Hubbard system by taking a next-nearest-hopping into account^9^. In the Mott insulating systems, relatively strong magnetic interactions with *J/k*_B_ = −10^2–4^ K originating from the on-site and the inter-site Coulomb correlations are playing substantial roles^10,11^. The hole/electron carriers introduced by elemental substitutions, intercalations of anion/cation species between the two dimensional (2D) layers, or the gate-bias in field-effect-transistor (FET) structures can make carrier doped situations and induce anomalous electronic states^12,13^. Since the strong antiferromagnetic interaction is certainly essential in the doped Mott physics, ferromagnetic features reported so far occur only collaterally as a spin canting through Dzyaloshinsky–Moriya (DM) interactions or double exchange mechanism in manganese oxides^3,14^. The realization of the bulk ferromagnetism predicted by the theoretical models in Refs.^7–9^ has not been succeeded up to now. By using effects of the electrostatic potential due to net polarization *P* of counter-ion layers in molecular charge transfer complexes, carrier control in extremely small amounts to change the electronic states of π-electrons can be possible as is reported several charge transfer complexes. It may give an appropriate stage for investigating doping ferromagnetism in Mott–Hubbard systems.

**Figure 1:**
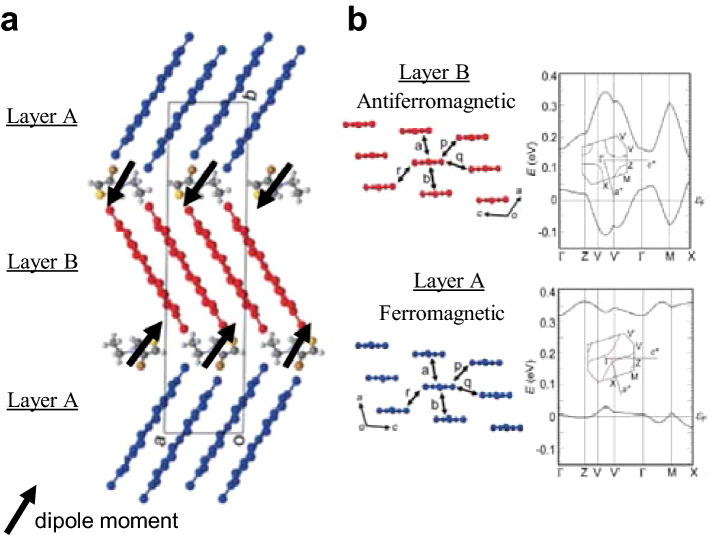
The crystal structure and the electronic band structure of bi-layer compound of (Et-4BrT)[Ni(dmit)_2_]_2_. (**a**) The crystal structure and the counter cations alignment. The polarized cations are aligned in a direction shown by black arrows to make different dimer stacking in the layer A and the layer B. (**b**) Molecular arrangement of the Ni(dmit)_2_ molecules and the band structures of the two types of layers in (Et-4BrT)[Ni(dmit)_2_]_2_. The values of overlap integral (× 10^–3^) of each layer is *a* = − 17.1, *b* = 0.15, *p* = − 0.05, *q* = 0.41, *r* = 1.88 for the layer A and *a* = 10.6, *b* = 3.96, *p* = − 5.50, *q* = 0.46, *r* = − 2.63 for the layer B. The degree of dimerization is rigid in the Layer A, while that of Layer B is rather moderate. The molecular packing and band dispersion in each layer are adapted with permission from (Ref.^17^, T. Kusamoto et al. *Inorg. Chem.* **51**, 11645–11654 (2013)). Copyright (2013) American Chemical Society.

**Figure 2:**
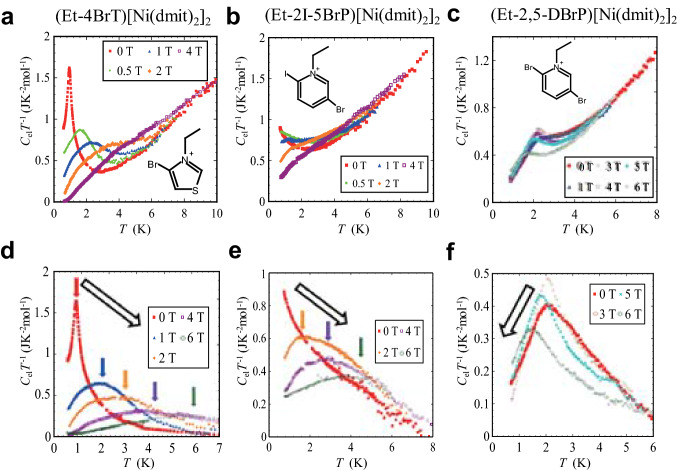
The temperature and the magnetic field dependences of the heat capacities of the series of bi-layer compounds. Temperature dependences of heat capacity of three bi-layer compounds of (**a**) (Et-4BrT)[Ni(dmit)_2_]_2_, (**b**) (Et-2I-5BrP)[Ni(dmit)_2_]_2_, and (**c**) (Et-2,5-DBrP)[Ni(dmit)_2_]_2_. The magnetic heat capacities are shown in the same plot. The electronic contributions of the specific heat obtained by subtracting the lattice contributions for (**d**) (Et-4BrT)[Ni(dmit)_2_]_2_, (**e** (Et-2I-5BrP)[Ni(dmit)_2_]_2_, and (**f**) (Et-2,5-DBrP)[Ni(dmit)_2_]_2_ are also shown. The colored arrows show the peak position of the magnetic heat capacity and the black arrows show the tendency of the peak shift with the increase of the magnetic field. The tendency in (Et-4BrT)[Ni(dmit)_2_]_2_ and (Et-2I-5BrP)[Ni(dmit)_2_]_2_ demonstrates that the ferromagnetic features exist.

The materials we focus on in this work are bi-layer type dimer-Mott insulators with chemical formula of X[Ni(dmit)_2_]_2_ (dmit = 1,3-dithiole-2-thione-4,5-dithiolate). These compounds are consisting of the acceptor molecule of Ni(dmit)_2_ and mono-valent organic counter cation X with polarization. The crystal structure is shown in Fig. 1. Among numerous anion radical compounds with M(dmit)_2_ (M = Ni, Pd, Pt), a peculiar structural aspect of the present system is the ordered arrangement of asymmetric cations to make their dipole moments parallel and to produce net polarization (*P*) between the acceptor layers^15–17^. The electrostatic potential due to large *P* affects the Ni(dmit)_2_ layers where π-electrons show strong antiferromagnetic correlations. According to the preceding works by Kusamoto et al., the direction of the cation dipole moments is alternated oppositely between the cation layers. Consequently two types of Ni(dmit)_2_ layers, denoted as A and B in the figure, with slightly different molecular packings are realized^16,17^. The degree of dimerization and the band structure are different between the two layers as is shown in Fig. 1. In contrast with the antiferromagnetic correlation between the spins in the layer B of (Et-4BrT)[Ni(dmit)_2_]_2_, where Et-4BrT is ethyl-4-bromothiazolium, the spins in the layer A keep paramagnetic state with ferromagnetic correlation. They reported that long-range magnetic ordering does not appear down to 2 K^16,17^. In the inset of Fig. 2a–c, we show the molecular structure of the counter cations of X = Et-4BrT, ethyl-2-iodo-5-bromopyridinium (Et-2I-5BrP), ethyl-2,5-dibromopyridinium (Et-2,5-DBrP), where the magnitude of dipole moments are different depending on the molecular structure. The net polarization of the counter cation layers forms electrostatic potential to the π-electrons layers. The heat capacity, magnetization, and ESR measurements of (Et-4BrT)[Ni(dmit)_2_]_2_ show evidence of a long-range magnetic order with ferromagnetic character at 1.0 K. We also observed a systematic variation of the ground state from antiferromagnetic one to ferromagnetic one with the increase of *P* tunable by using difference of counter cations.

## Results

**Figure 3:**
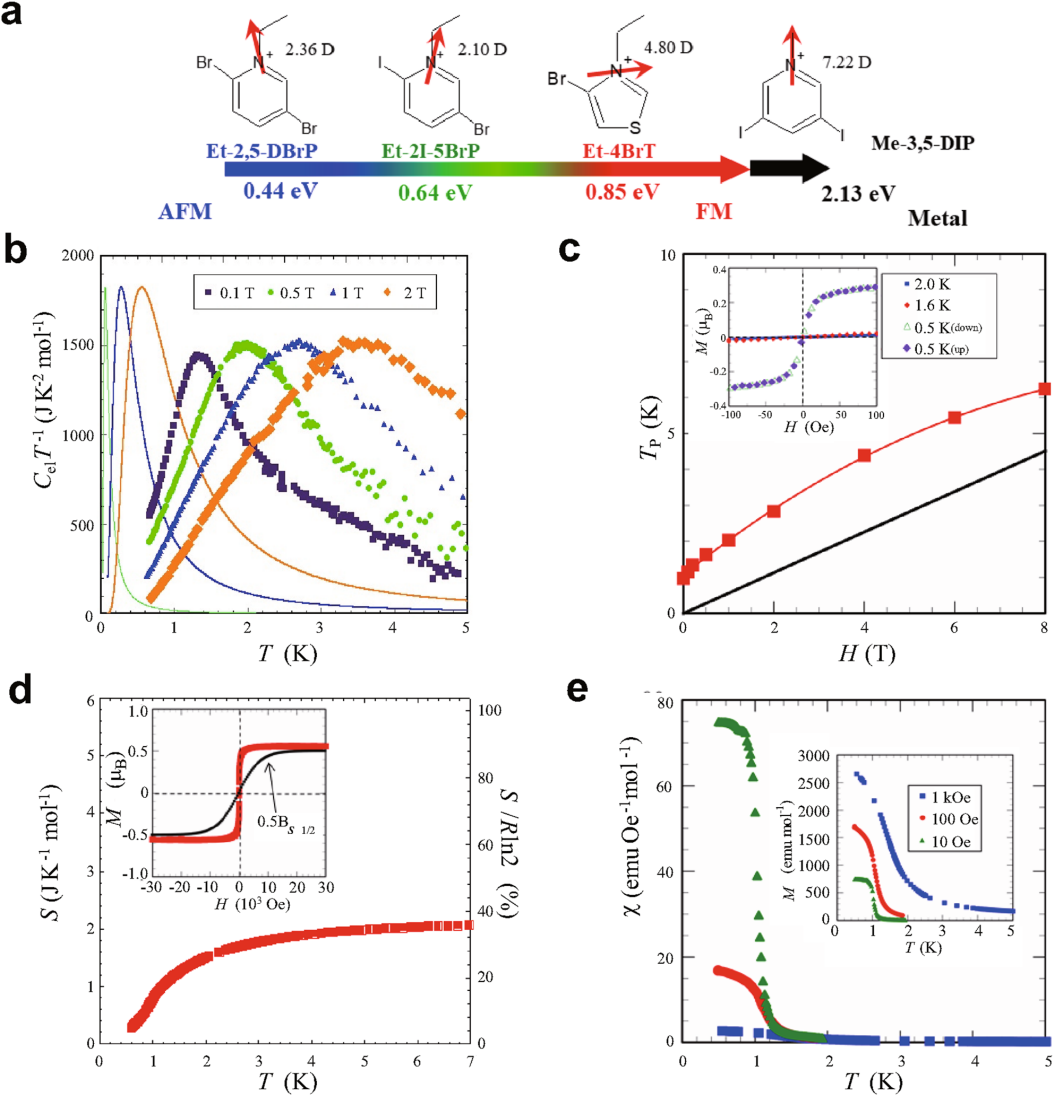
Molecular structures of counter cations of the series of bi-layer compounds and magnetic and thermal properties of (Et-4BrT)[Ni(dmit)_2_]_2._ (**a**) The schematic view of the molecular structure of polarized cations. The red arrows indicate the direction of the dipole moments, and the calculated electrostatic potential values in the layer A produced by the aligned cations are given in the figure. (**b**) The electronic heat capacity under magnetic field of 0.1 T, 0.5 T, 1.0 T and 2. 0 T (filled circles), compared with the simulated Schottky heat capacity under the same magnetic fields (0.5 T, 1.0 T, and 2.0 T) (solid lines). (**c**) Magnetic-field dependence of the heat capacity peak (*T*_*p*_) compared with the paramagnetic case represented by Schottky heat capacity (black line). The inset shows the magnetization curves between -100 Oe and 100 Oe at 0.5 K, 1.6 K, and 2.0 K. (**d**) Temperature dependence of magnetic entropy of (Et-4BrT)[Ni(dmit)_2_]_2_ derived from the magnetic heat capacity. The inset shows the magnetization curve at 0.5 K which shows ferromagnetic saturation of the magnetization. The black solid curve in the inset is the Brillouin function expected for the case of paramagnetic case. (**e**) Temperature dependence of magnetic susceptibility of (Et-4BrT)[Ni(dmit)_2_]_2_.The inset shows the temperature dependence of magnetization. The external fields are 10 Oe, 100 Oe, and 1 kOe.

The results of low temperature heat capacity measurements of (Et-4BrT)[Ni(dmit)_2_]_2_ of which counter cations have relatively large dipole moment with 4.80 D are shown in Fig. 2a. The measurements were performed for several pieces of single crystals with the total weight of 240 μg. A sharp peak in the heat capacity in association with the magnetic transition was observed at extremely low temperature region around 1 K. Since the π-electrons in the acceptor layers are localized over each Ni(dmit)_2_ dimer, it is considered as a long-range ordering of π-electron spins with *S* = 1/2. The magnetic field dependence shown in the same figure demonstrates that the magnetic order has a feature of bulk ferromagnetic character, since the application of magnetic field makes the sharp peak broaden and upward shifts the peak temperature. The appearance of the sharp peak in the heat capacity at *H* = 0 T and the feature under magnetic fields resemble well with that of the β-phase of *p*-NPNN (*para*-nitrophenylenitronylenitroxide) known as bulk ferromagnet of organic radical spins^18,19^. The temperature and the magnetic field dependences of the heat capacities of two other compounds with less asymmetric cations X = Et-2I-5BrP and Et-2,5-DBrP are compared in Fig. 2b,c. (Et-2I-5BrP)[Ni(dmit)_2_]_2_ shows an increase of *C*_*p*_ below 2 K and (Et-2,5-DBrP)[Ni(dmit)_2_]^2^ shows a peak at 0 T. The lattice heat capacity of these magnetic compounds are evaluated based on the data of similar bi-layer compound (Me-3,5-DIP)[Ni(dmit)_2_]_2_, where Me-3,5-DIP is methyl-3,5-diiodopyridinium, which have non-magnetic and the metallic character in this temperature region. The details are given in supplementary information. The magnetic heat capacities obtained by subtracting the lattice contributions for (Et-4BrT)[Ni(dmit)_2_]_2_, (Et-2I-5BrP)[Ni(dmit)_2_]_2_, (Et-2,5-DBrP)[Ni(dmit)_2_]_2_ are also shown in Fig. 2d, e, and f, respectively. We could not observe a peak structure in the experimental region down to 0.7 K for X = Et-2I-5BrP as is shown in Fig. 2e but the magnetic field dependence distinctly suggests that the transition should exist at lower temperatures. The emergence of the broadened peaks under magnetic fields above 1 T for (Et-2I-5BrP)[Ni(dmit)_2_]_2_ reflects the entropy shift to the higher temperature side by applying magnetic fields just as the case for (Et-4BrT)[Ni(dmit)_2_]_2_. This result implies that the ground state of this compound is also ferromagnetic state. On the other hand, (Et-2,5-DBrP)[Ni(dmit)_2_]_2_ shows a complicated magnetic field dependence. The change of peak shape of *C*_*p*_ by applying magnetic field reveals that the magnetic transition is an antiferromagnetic one, since the peak shape keeps sharp and shifts to lower temperature in the higher magnetic field region above 3 T. However, data at the lower fields show complicated behavior demonstrating that the ferromagnetic and the antiferromagnetic components compete each other in the compound. The difference of the magnitude of dipole moments of counter cations and possible electrostatic energy induced in the acceptor layers are shown in Fig. 3a.

The peak shape of the magnetic heat capacity, *C*_el_ at 0.1 T, 0.5 T, 1.0 T and 2.0 T obtained by subtracting the lattice part shown in Fig. 2d are compared with the Schottky peaks in the same magnetic fields in Fig. 3b. The broadened peak shape of the present compound under magnetic fields is different from the typical Schottky anomalies. In Fig. 3c, we also show the magnetic field dependence of the peak temperature, *T*_p_ of heat capacity divided by temperature of (Et-4BrT)[Ni(dmit)_2_]_2_. The shift of the peak is larger than that expected for the Schottky heat capacity expected for the Zeeman splitting of *S* = 1/2 paramagnetic spins. These results also support the ferromagnetic ground state of this compound. The temperature dependence of the magnetic entropy evaluated by subtracting lattice heat capacity is shown in Fig. 3d. Although the absence of data at the lowest temperature region below 0.6 K contains ambiguity, it reaches to 74% of 1/2*R*ln2 at 6 K which means that nearly half spins are involved in the long-range ferromagnetic ordering.

The formation of the ferromagnetic long-range order can also be confirmed from the magnetization measurements in the ^3^He temperature region. Figure 3e shows the temperature dependency of the magnetic susceptibility of (Et-4BrT)[Ni(dmit)_2_]_2_ in a* χ* versus *T* plot. Those of magnetization per formula unit are shown in the inset of Fig. 3e. The data obtained at 1 kOe reproduces well the already published data obtained by SQUID measurements above 2 K^17^. The previous data by Kusamoto et al. in Ref.^17^ have suggested a small ferromagnetic component at low temperatures, although the antiferromagnetic-type interactions in Mott insulating states that are dominant at high temperature region. Kusamoto et al*.* reported that the antiferromagnetic component is arisen from relatively strong interaction in the layer B and the ferromagnetic one comes from the interactions of localized spins in the layer A. From the low temperature susceptibility and the magnetization between 0.5 K and 2 K obtained at weak field of 10 Oe, 100 Oe, and those obtained between 0.5 K and 5 K at 1kOe shown Fig. 3e, we can notice of a drastic increase of the magnetization around 1 K corresponding to the temperature of the sharp peak in the heat capacity data. This result ensures that the bulk ferromagnetic transition certainly occurs in this compound. The magnetization versus magnetic field (*M*-*H*) curve at the lowest temperature of 0.5 K plotted in the inset of Fig. 3d shows an abrupt increase of the magnetization at the low-field region and saturation above 500–1000 Oe. The increase is sharper as compared with the feature of the Brillouin function for *S* = 1/2 especially in the low field region. In the inset of Fig. 3 c we show the *M–H* curves between − 100 Oe and 100 Oe obtained at 0.5 K, 1.6 K, and 2.0 K in the same plot. The *M–H* curves at paramagnetic region of 1.6 K and 2.0 K show almost linear and have smaller absolute values of *M*. However, that obtained at 0.5 K show an abrupt increase of *M* in the low field region below ± 100 Oe characteristic of the ferromagnetic materials. The hysteresis cannot be observed within the experimental resolution of ± few Oe. Since the magnetic field in this experiment is applied by a superconductive magnet, we do not have enough resolution for the lower field region in the present set up. In the case of *p*-NPNN, the magnitude of the hysteresis is smaller than ± 10 Oe^19^. Another organic ferromagnet, TDAE-C_60_, where TDAE is tetrakis(dimethylamino)ethylene do not show any hysteresis despite that its *T*_c_ is 16 K^20^. Relatively soft ferromagnetic in the present compound seems to be a common feature for organic ferromagnets.

The saturation value of the magnetization at 0.5 K shown the inset of Fig. 3d reaches to 0.5 *μ*_B_ per Ni(dmit)_2_ dimer, which means that nearly half of *S* = 1/2 spins are involved in the ferromagnetic ordering. This is consistent with the feature of magnetic entropy of the spins shown in Fig. 3 d. Considering the low dimensional short-range feature existing at higher temperature than *T*_c_, the value of the entropy of 74% of 1/2*R*ln2 at 6 K is consistent with the model that the nearly half of π-electron spins in the whole crystal are related to the transition. The consistency of the saturation value of the magnetization and the spin entropy means that the ferromagnetic order of π-electron spins occurs either one of the two layers, probably in the layer A, of the bi-layer compound. The magnetic entropy in other two compounds also reveals the bulk nature of the transition by analyzing the heat capacity data shown in Fig. 2b,c. The temperature dependences of magnetic entropy for these compounds are shown in Fig. S3 in the supplementary information.

**Figure 4:**
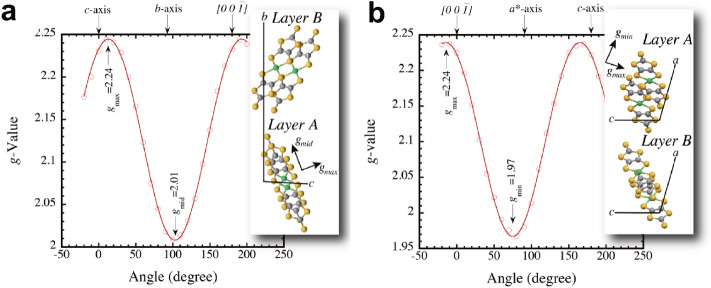
The angular dependence of the *g*-value. The *g*-value anisotropies of (Et-4BrT)[Ni(dmit)_2_]_2_ in the layer (**a**) *bc*-plane and (**b**) a*c-plane measured from the angular dependence of ESR at 4 K. The a*-axis is the axis perpendicular to the *bc*-plane. The insets show the relations between the observed principal axes of the *g*-tensor and the molecular arrangements of the dimer in layer A and B. The principal axes (*g*_max_, *g*_mid_ and *g*_min_) coincide with the short, long and vertical axes of the dimer in layer A, respectively.

In order to confirm the origin of the nearly one-half magnetization and the spin entropy, electron spin resonance (ESR) measurements using a single crystal are performed at 4 K. The ESR signal of the Ni(dmit)_2_ dimer with *S* = 1/2 generally shows an anisotropic *g*-value where the principal axes of the *g*-tensor coincide with the molecular axes of the dimer. The angular dependence of *g*-value in the *bc*- and *a* * *c*-planes are shown in Fig. 4a,b respectively. A relatively large anisotropy of the *g*-value is observed for each plane, and the principal axes of the *g*-tensor corresponds well with the dimer arrangement of layer A. These results clearly confirm that the spin origin of the low temperature ferromagnetism is from the layer A and not from layer B.

## Discussion

The realization of bulk ferromagnetic transition in the dimer-Mott compounds is unusual in terms of electron correlations, since they usually have rather strong antiferromagnetic interaction dominated by the on-site Coulomb repulsion in 2D or quasi-1D structure. The topics related to the unconventional superconductivity, spin liquids^21–23^, and the quantum charge glasses^24^ etc. produced by π-electrons in organic charge transfer complexes are dominated by antiferromagnetic interactions in Mott–Hubbard systems. The ferromagnetic moments accompanied by spin canting in the antiferromagnetic ordering was observed in κ-(BEDT-TTF)_2_Cu[N(CN)_2_]Cl^25^, but its magnetization value is quite small and is usually less than 0.05 *μ*_B_ while entropic contribution for ferromagnetic ordering is almost negligible. This is in fine contrast with the present case where bulk transition ensured by entropic analysis as is in radical magnets certainly occurs. We believe that the bulk ferromagnetic transition observed here can be considered as a manifestation of the subtle doping effect in dimer-Mott insulators. The important factor is the net polarization in the cations layers as was compared in Fig. 3a. The polarization *P* directed towards the layer B from both sides can produce a kind of hole-doped and electron-doped situations in the layers A and B, respectively, as is realized in FET devices. Such an electric-field-effect provided by the molecular dipole moment has been utilized for photo-induced superconducting transition^12,13^. The carrier control for the surface area of organic Mott insulating state was realized by the fabrication of FET structure in a typical dimer-Mott compound of κ-(BEDT-TTF)_2_X, and they reported the appearance of doping-induced superconductivity^12^. The unusual transport features probably related to the polarization induced doping in the similar-type bi-layer compounds with TTF analogous donors were also reported for (TTF)_3_(PO-CON(CH_3_)C_2_H_4_SO_3_)_2_^26^, κ_H_-(DMEDO-TSeF)_2_[Au(CN)_4_](THF)^27^, and κ-β”-(BEDT-TTF)_2_PO-CONHC_2_H_4_SO_3_^28^. In the latter two bi-layer compounds, the value of charge imbalance between two different layers were evaluate as Δ*ρ* = 0.025, and Δ*ρ* = 0.08 from the size of Fermi surface detected by Shubnikov-de Haas oscillation analyses.

The quantitative comparison of the magnitude of dipole moments and electrostatic field produced by the counter cations for the present systems are shown in Fig. 3a. (Et-4-BrT)[Ni(dmit)_2_]_2_ has largest dipole moment of 4.80 D that produces an effective electrostatic potential of 0.85 eV for the layer A according to the calculation method by Suda et al.^29^ The value decreases systematically from (Et-4-BrT)[Ni(dmit)_2_]_2_ to other two compounds with less asymmetric structures in Et-2I-5BrP^+^, Et-2,5-DBrP^+^. Although the latter two cations have almost similar dipole moments, the calculated electric field taking account of the polarization direction gives the values shown in Fig. 3a which is consistent with the magnetic feature in the π-electron layers. The doping level in the present compounds are evaluated as 2.1% for Et-4-BrT compound, 1.6% for Et-2I-5BrP, and 1.1% for Et-2,5-DBrP compound referring the charge imbalance values in Ref.^28^. We assumed that the magnitude of electrostatic potential calculated from the dipole moment of the counter ions are proportional to the doping rate. The compounds reported in Ref.^28^ has much larger dipole moments and consequently the electrostatic potentials to make charge imbalance are reported as 3.2 eV and 7.7 eV which is one order larger than the current complexes. The metallic conductivity in the layers may also give different situation from the present system. Furthermore, the Coulomb screening due to the delocalized character of π-electrons in the molecular orbitals is not taken into accounts in this calculation and therefore the values of the doping rate may be overestimation. The direct evaluation of the exact rate is required. However, the existence of small doping and systematic change of the physical properties appears concomitantly with the magnitude of electrostatic potential should be intrinsic for this system. The decrease of the polarization results in the suppression of ferromagnetic behavior and the ground state changes to the original antiferromagnetic feature as in the case of X = Et-2,5-DBrP compound.

It is known that small amount of carrier doping in Mott insulating system may produce a kind of ferromagnetic feature around the electrons/holes as is discussed in extremely light doped region of La_2−x_Sr_x_CuO_4_ (LSCO) system^30^. However, the carrier doping induced by the substitution of metal cations with different valences and sizes produces a local disorder to make a kind of glassy state. It is really the case in spin glass region of LSCO where the net antiferromagnetic interaction is disordered by the appearance of local ferromagnetic interactions. The ordered arrangement of the polarized cations in the present compounds can, on the other hand, produce homogenous electric fields in the Mott insulating layer and induce such a drastic ground state change in a bulk level, although the doping amount may be extremely small in this case. Theoretically predicted ferromagnetism of Nagaoka^7^ and Penn^8^ seems to be applicable in this quite lightly doped situation. These models treated an extreme case and their realization has been believed to be difficult in real materials, because the replacement of constitutive elements or ions always induce potential disorder and spin-glass features as in the case of LSCO^30^. However, the net polarization effect from the counter cation layers can realize homogeneous influence throughout the π-electron layer. The molecular hybrid system like the present bi-layer compounds may give suitable stage for attaining the situation expected for Nagaoka–Penn ferromagnetism. The extremely soft nature of the ferromagnet suggests an absence of apparent magnetic anisotropy, which may be related to the quantum nature of such ferromagnetism.

**Figure 5:**
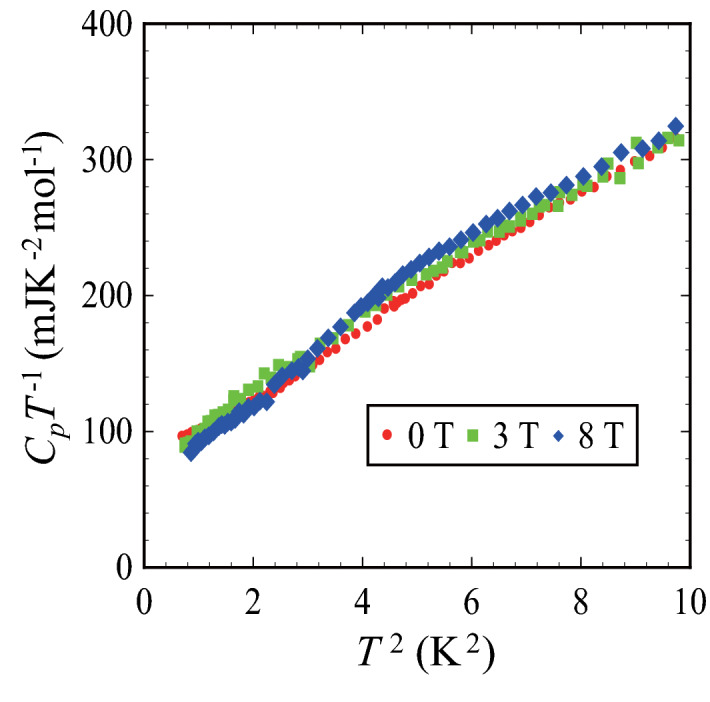
Temperature and magnetic field dependences of the heat capacity of (Me-3,5-DIP)[Ni(dmit)_2_]_2_. Temperature dependences of the heat capacity of (Me-3,5-DIP)[Ni(dmit)_2_]_2_ obtained at 0, 3, 8 T shown in *C*_*p*_*T* ^−1^ versus *T* ^2^ plot. Owing to the increase of doping, the compound shows typical metallic feature expressed by the electric contribution (*γT* ) and the lattice contribution (*βT* ^3^).

In accordance with this doping scenario, it is expected that the further doping by utilizing counter cations with larger dipolar moments can change the ferromagnetic ground state to a metallic state. As a matter of fact, in (Me-3,5-DIP)[Ni(dmit)_2_]_2_ of which counter cations have larger dipole moment of 7.22 D as is shown in Fig. 3a gives a metallic ground state^15^. The alignment of this large dipole moment induces an electronic potential of 2.13 eV which is 2.5 times larger than 0.85 eV for (Et-4-BrT)[Ni(dmit)_2_]_2_. The increase of the net polarization induces mobile carriers in the Mott insulating layer, and the system transforms into a metallic state in effectively half-filled states. The results of the heat capacity measurements of (Me-3,5-DIP)[Ni(dmit)_2_]_2_ shown in Fig. 5. This compound does not show long-range magnetic transition but gives typical feature of metallic system. The *C*_*p*_*T*^−1^ versus *T*^2^ plot shows a linear relation with a finite electronic heat capacity coefficient *γ* at low temperature region. The *γ* value determined by linear extrapolation gives an anomalously large value of 60 mJK^−2^ mol^−1^. Kosaka et al. claimed in Ref.^15^ that one layer retains Mott insulating character with localized spins but the other layer gives conductive properties in this compound. Considering this situation, the effective *γ* of the metallic layers should be twice as large as the experimental value which is extraordinary larger as molecular conductors. The value is larger than *γ* = 55 mJK^−2^ mol^−1^ of κ-(BEDT-TTF)_4_Hg_2.78_Cl_8_ which gives probably the largest *γ* among κ-type metallic compounds^31^. It is natural to connect this larger electronic heat capacity to the ferromagnetic spin fluctuations which enhances the electronic heat capacity as is discussed in intermetallic compounds such as ScIn_3_, ZrZn_2_ etc.^32^ The magnetic fields dependence observed in rather high magnetic fields of 3 T and 8 T can be explained by the fluctuations of itinerant electrons.

**Figure 6:**
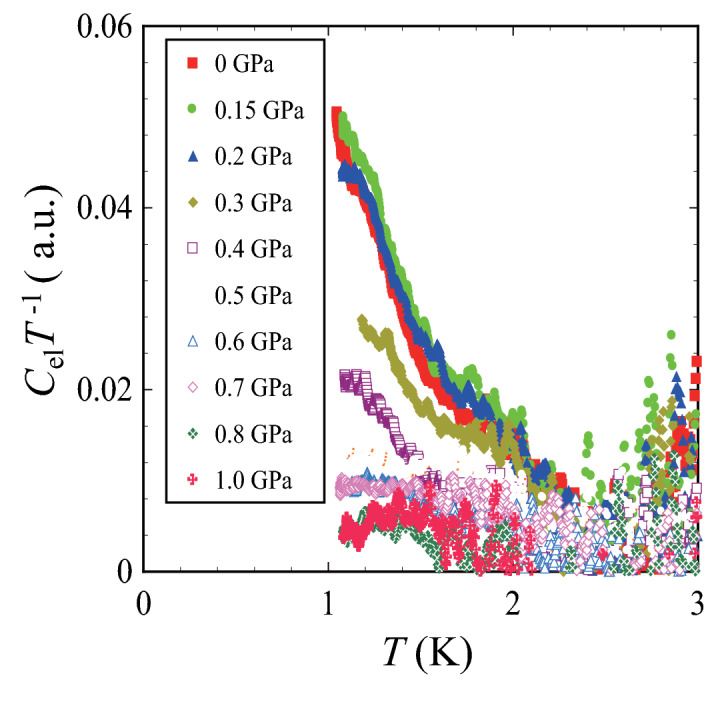
Pressure dependence of the heat capacity peak of (Et-4BrT)[Ni(dmit)_2_]_2_. Pressure dependence of the heat capacity of (Et-4BrT)[Ni(dmit)_2_]_2_ obtained under pressure up to 1.0 GPa. The magnetic ordering of localized π-electrons on dimers is suppressed by pressure and gradually changes to the metallic ground state with the increase of external pressure.

Since the bulk ferromagnetic state of (Et-4-BrT)[Ni(dmit)_2_]_2_ is realized by the extremely light doping in a Mott insulating state, it should gradually change into an itinerant electronic state also by applying pressure through the band width control mechanism. In fact, Kusamoto et al. performed resistivity measurements under various external pressures and reported gradual appearance of conductive properties as is reported in the supplementary information of Ref.^17^. This feature is reproduced in the present sample also as is shown in the Fig. S10 in the supplemental information of this paper. The ground state in the high-pressure region gradually changes from insulating state to conductive state. To confirm the phase relation around the ferromagnetic ground state in the Mott insulating region, high-pressure heat capacity measurements for (Et-4-BrT)[Ni(dmit)_2_]_2_ were performed by low temperature ac calorimetry technique for a pellet sample of 230 μg. The higher temperature tail of the heat capacity peak was clearly observed by the high-pressure calorimeter at ambient pressure as is shown in Fig. 6. We observed the peak structure is quite sensitive to the external pressure, and it is gradually suppressed with the increase of pressure. Results of external pressure dependences of the heat capacity measurements demonstrate that the ferromagnetic state is certainly suppressed by hydrostatic pressure. Above 1.0 GPa, the system turns into a non-magnetic state without any thermal anomaly within the experimental resolution and have finite *γ* term because it gives constant shift in *C*_el_*T* ^−1^. This feature is consistent with the transport measurements in that the hydrostatic pressures higher than 1.0 GPa leads the system to a metallic state^17^. It should be mentioned that the changes in electronic feature produced by the carrier doping and the pressure effects should be different, but the results of the high pressure heat capacity certainly demonstrate that the system is Mott insulating state located very close to the anomalous metallic phase as in organic dimer-Mott compounds. The feature of conductive states should contain further fascinating possibility to give ferromagnetic fluctuations in metallic states.

## Summary

In summary, we report the discovery of a novel type of ferromagnet in the bi-layer π-electron Mott insulating compounds with polarized cations from the results of heat capacity, magnetization, and ESR measurements. Since the molecular ferromagnets are usually designed by adjusting the overlap of molecular orbitals according to the McConnel’s mechanism as was realized in *p*-NPNN and other radical-based compounds^33^, the present mechanism may give new strategy for designing ferromagnets in molecular compounds. The subtle but homogeneous doping through polarization of cation-layer can give new approach not only in designing ferromagnets but also in designing superconductor with doped-Mott compounds. It may also lead to further possibility to produce magnetic superconductor where ferromagnetic fluctuations are important.

## Methods

### Preparation of single crystal samples

The single crystals of series of the bi-layer anion radical salts, X[Ni(dmit)_2_]_2_ (X = Et-4BrT, Et-2I-5BrP, Et-2,5-DBrP, and Me-3,5-DIP ), were synthesized according to the procedure reported in Refs.^15–17^. It was confirmed that the crystals obtained in the same batch show same physical properties reported in the references.

### Heat capacity measurements

Low-temperature heat capacity measurements were performed by a thermal relaxation calorimeter constructed for measuring single crystal samples weighing about 10^1–3^μg. The system is custom-made one developed by the Osaka university group. The heat capacity under applied pressures were performed by ac calorimetry technique using a standard clump type pressure cell made of Cu-Be alloy with dapheni 7373 oil as a pressure medium. The details of the apparatus and analytic methods are given in the supplementary information.

### ESR measurements

ESR measurements using a single crystal were performed by a conventional X-band ESR spectrometer (JEOL JES-RE3X) equipped with a He-flow cryostat (Oxford Instruments). The sample was mounted on a quartz rod using a silicone grease, and the angular dependence of ESR was performed by rotating the quartz rod in the vertical direction of the applied magnetic field.

## Supplementary information

Supplementary Information.
