# Supplementary material for: Electric dipole induced bulk ferromagnetism in dimer Mott molecular compounds
Source: Sci Rep. 2021 Jan 14;11:1332. doi: 10.1038/s41598-020-79262-6 (PMC7809364; doi:10.1038/s41598-020-79262-6)
Supplement: Supplementary file 1 — Supplementary Information. [file 41598_2020_79262_MOESM1_ESM.doc]

Supplementary Information

Electric Dipole Induced Bulk Ferromagnetism in

Dimer Mott Molecular Compounds

Ryo Yoshimoto^1^, Satoshi Yamashita^1^, Hiroki Akutsu^1^, Yasuhiro Nakazawa^1*^, Tetsuro Kusamoto^2^, Yugo Oshima^3^, Takehito Nakano^4^, Hiroshi M. Yamamoto^5^, and Reizo Kato^3^

^1^*Department of Chemistry, Graduate School of Science, Osaka University, Toyonaka, Osaka, 560-0043, Japan*

^2^*Department of Life and Coordination-Complex Molecular Science, Institute for Molecular Science, Okazaki, Aichi, 444-8787 Japan*

^3^ *RIKEN, Cluster for Pioneering Research (CPR), Wako, Saitama 351-0198 Japan*

^4^ *Institute of Quantum Beam Science, Graduate School of Science and Engineering, Ibaraki University, Mito, Ibaraki, 310-8512, Japan*

^5^ *Research Center of Integrative Molecular Systems (CIMoS), Institute for Molecular Science, Okazaki, Aichi 444-8585 Japan*

^*^Corresponding and requests for materials should be addressed to Y.N. (email: [nakazawa@chem.sci.osaka-u.ac.jp](mailto:nakazawa@chem.sci.osaka-u.ac.jp))

The low temperature heat capacity measurements for (Et-4BrT)[Ni(dmit)_2_]_2_, (Et-2I-5BrP)[Ni(dmit)_2_]_2_ , (Et-2,5-DBrP)[Ni(dmit)_2_]_2_ and (Me-3,5-DIP)[Ni(dmit)_2_]_2_ were performed by the thermal relaxation calorimetry technique using an original calorimeter designed for measuring heat capacity of small amount of samples weighing 10^1-3^ μg. The schematic illustration of the calorimetry cell used for these experiments is shown in Fig. S1 **a, b**. This calorimetry cell is consisting of a ruthenium oxide sensor of which room temperature resistance is 1.0 kΩ.  We also used a strain gauge resistance with 1.0 kΩ as a sample heater of the calorimetry cell. The thermal link between the sample stage and the heat sink was borne by thin constantan wires with a diameter of 13 μm which also used as electric leads for the thermometer and the heater. The photo picture of the typical single crystal of (Et-4BrT)[Ni(dmit)_2_]_2_ used in this experiments are presented in Fig. S1 **c**. The typical Δ*T* values for the temperature relaxation process were about 20-60 mK depending on the temperature region and the relaxation time of the sample measurements was in the range of 10^0-2^ sec. Details of the relaxation calorimetry systems are given in the literatures. (R1,R2)

Figure S1 **a, b** A schematic view and a photo picture of the relaxation calorimetry cell used for the experiments. **c** A photo picture of the typical single crystals of (Et-4BrT)[Ni(dmit)_2_]_2_.

The overall temperature dependence of the heat capacity of (Et-4BrT)[Ni(dmit)_2_]_2_ between 0.6 K and 50 K is shown in *C_p_T* ^-1^ vs *T* plot in Fig. S2 **a** using a semi-logarithmic scale of temperature. The heat capacity of (Et-4BrT)[Ni(dmit)_2_]_2_ shows smooth temperature dependence except for the sharp anomaly corresponding to the long-range ordering of π-electron spins. The lattice heat capacity is evaluated based on the data of similar bi-layer compound (Me-3,5-DIP)[Ni(dmit)_2_]_2_ which have the metallic character. From the *β* coefficient of the *T* ^3^ term corresponding to the Debye approximation, it is possible to evaluate the lattice contribution of the heat capacity. The experimental data of the latter compound is shown as the black dots in Fig. S2 **b**. Using the *β *value of this metallic compound, we evaluated the possible lattice heat capacity of (Et-4BrT)[Ni(dmit)_2_]_2_ which are shown in the red dashed curve in the same figure. The magnetic heat capacities shown in Fig. 2 in the main text are obtained by subtracting the lattice heat capacities for each compound. The temperature dependences of the magnetic entropy of the three compounds are shown in Fig.S3. Here we assume that the temperature dependence of low temperature heat capacity below 0.6 K obeys the model of the spin-wave excitations. The magnetic entropy of (Et-2I-5BrP)[Ni(dmit)_2_]_2_ contains some ambiguity (within ±10%), since *C_p_T* ^-1^ is continuously increasing with the decrease of temperature due to the possible ordering and the extrapolation by the spin-wave model is not possible.

Figure S2 **a** Temperature dependence of the heat capacity of (Et-4BrT)[Ni(dmit)_2_]_2_ in log scale of temperature. **b** The dash line shows the lattice heat capacity of (Et-4BrT)[Ni(dmit)_2_]_2_ evaluated by subtracting the electronic heat capacity *γ* from the heat capacity of (Me-3,5-DIP)[Ni(dmit)_2_]_2_ shown as black dots.

Figure S3 Temperature dependences of the spin entropy of three bi-layer compounds showing the magnetic orderings.

Figs.S4 **a, b** shows the schematic illustration of the sample set up of the high-pressure calorimetry and the photo picture of sample part of which details were reported previously. (R3, R4) The pellet sample with total weight of 0.230 mg was sandwiched by two chip type sensors used as a thermometer and a heater. The temperature oscillation amplitude, Δ*T*_ac_ induced by applying constant currents can be detected by lock-in amplifier. The frequency dependences of the ac oscillation amplitude are shown in Fig. S4 **c**. The frequency of 20.8 Hz indicated by a dashed line in the figure was selected as the measurement frequency located in the plateau region of *ω*Δ*T* _ac_ between internal time constant *τ*_in_ and external time constant *τ*_ext_.

Figure S4 **a** A schematic view of high-pressure ac calorimetry cell and the sample part. **b** A picture of the sample part consisting of crystals and the thermometer and the heater chips. **c** Frequency dependences of the temperature modulation amplitude at various frequencies of (Et-4BrT)[Ni(dmit)_2_]_2_ in the high pressure ac calorimeter at ambient pressure.

In Fig. S5 **a**, temperature dependences of the total heat capacity of the sample part of the high-pressure calorimeter obtained by thermal relaxation method under magnetic fields between 0 T and 4 T are shown. The data contain addenda heat capacity consisting of those of the thermometer, the heater, and the epoxy utilized for ensuring good thermal contact between sample and chip type sensor and heater. Since the ac calorimetry performed in the pressure cell usually contains signal from large addenda heat capacity, we evaluated the temperature dependency of Δ*C_p_* defined as the discrepancy from the fitted curve based on the data under magnetic field of 4 T. The results were plotted in Δ*C_p_T* ^-1^ vs *T* in the figure. Since the magnetic field of 4 T is strong enough to suppress the heat capacity anomaly of the ferromagnetic transition due to the Zeeman splitting of the *S*=1/2 levels, the temperature dependences of Δ*C_p_T* ^-1^ reflect the relative change of the magnetic heat capacity of the sample. The magnetic fields dependences of the background contribution are not so large as (one or two order of magnitude smaller) compared with those of sample in the present localized spin system. The temperature dependence of Δ*C_p_T* ^-1^ of 0 T , 0.1 T, 0.2 T, 1.0 T and 2.0 T data are shown in Fig. S5 **b** together with the similar plot of Δ*C_p_T* ^-1^ =*C_p_T* ^-1^(*H*)-*C_p_T* ^-1^(4T) obtained for the measurement of relaxation calorimetry for the same sample with thermometer and heater.

Fig. S6 displays the pressure dependence of total heat capacity of the sample part at 0 T obtained by high pressure calorimeter. All heat capacity data with different pressures are plotted with a constant shift of the values of the vertical axis in order to compare the curve trend at a glance. In each pressure, we subtracted the data under magnetic field of 4T as background for evaluating magnetic heat capacity in lower fields region. The magnetic contributions in various pressures are plotted in Fig.4 **b** in the text to discuss the effect of external pressure for the ferromagnetic transition. Fig. S7 shows pressure dependences of heat capacity at 0.1 T, 0.2 T, 0.5 T and 1.0 T. In all magnetic fields, the external pressure works to suppress the magnetic heat capacity which means that the localized spins changes to the itinerant electrons which also support the information shown in Fig. 4 **b** in the main text.

Figure S5 **a** Temperature dependences of the total heat capacity of the sample part of the high-pressure calorimeter obtained by thermal relaxation method under magnetic fields. **b** Temperature dependence of Δ*C_p_T* ^-1^ = *C_p_T* ^-1^(*H*)-*C_p_T* ^-1^(4T) of 0 T, 0.1 T, 0.2 T, 1 T and 2T data.

Figure S6 Pressure dependency of the total heat capacity of the sample part at 0 T obtained by high pressure calorimeter.

Figure S7 Pressure dependences of the heat capacity at 0.1 T, 0.2 T, 0.5 T and 1 T.

The ESR measurements of (Et-4BrT)[Ni(dmit)_2_]_2_ at 4 K have been performed by using a conventional X-band ESR spectrometer (JEOL JES-RE3X, 9-10 GHz) equipped with a He-flow cryostat (Oxford Instruments). A quartz rod was used as a sample holder, and the rod was inserted in the cylindrical resonant cavity with TE_011_ mode. The magnetic field was applied horizontally to the quartz rod, and the angular dependence of ESR was performed by rotating the quartz rod. The error of the angle is within few degrees. The crystal axes of a single crystal were checked beforehand by X-ray diffraction measurement system (Rigaku HyPix-6000 AFC), and then, the sample was mounted on the quartz rod so that the magnetic field is applied parallel to the *bc*-plane or the *a***c*-plane. The applied microwave power for the ESR measurements was 4 mW, which is below the ESR’s saturation limit. The typical ESR spectra, when the magnetic field is applied parallel to the *bc*-plane and *a***c*-plane, are shown in Figs. S8 **a** and **b**, respectively. The ESR signal appears as a first derivative curve since the system uses the field modulation technique. Hence, the ESR’s resonance field *B*_res_ is taken from the intensity peak of the integrated ESR spectrum. Then, the g-value for each angle was deduced from the ESR’s resonance condition, *hν*=*gμ*_B_*B*_res_, where *h* is the plank’s constant, *ν* is the used microwave frequency, and *μ*_B_ is the Bohr magneton. The angular dependences of the *g*-value deduced from the spectra are shown in Figs. 4 **a** and **b** in the main text.


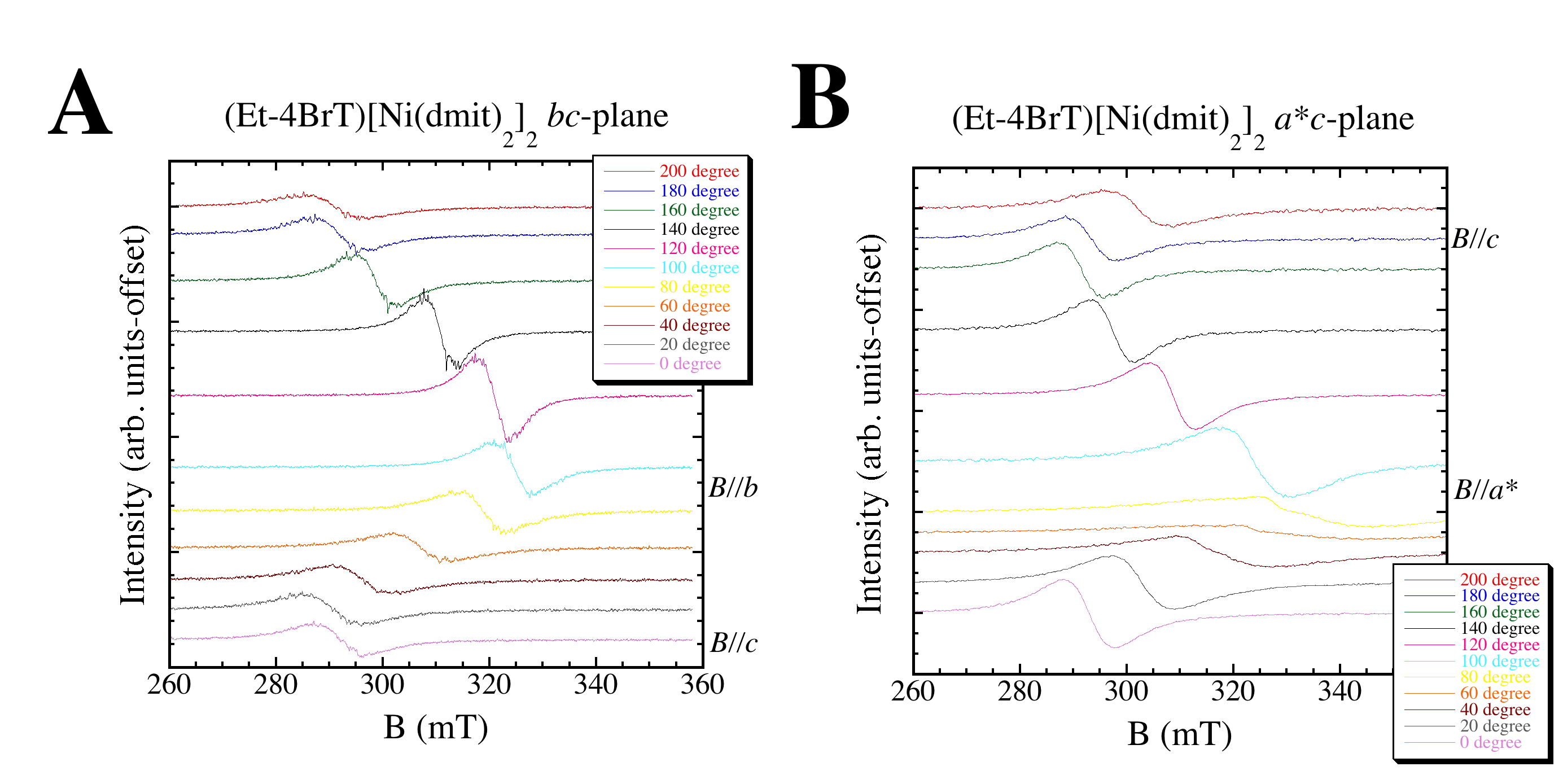


**a**

**b**

Figure S8 Typical ESR spectra of (Et-4BrT)[Ni(dmit)_2_]_2_ measured at 4 K for **a** *B*//*bc*-plane and **b** *B*//*a***c*-plane.

The magnetic field dependences of the heat capacity of (Et-4BrT)[Ni(dmit)_2_]_2_ obtained at higher temperature between 4 K and 50 K are shown in Fig.S9. The electron spins in layer B should give antiferromagnetic interactions below about 50 K and *χT* vs *T* plot show minimum around 30 K (Ref.17). Although the lattice contribution in this temperature region is relatively large, there are no signs of the magnetic transition. This result may be explained by the fact that magnetic entropy is distributed in wider temperature region due to the strong short-range fluctuations. These are typical feature of two-dimensional Mott insulators in the dimer-Mott compounds such as κ-(BEDT-TTF)_2_Cu[N(CN)_2_]Cl.

Figure S9 Temperature dependence of *C_p_T*^-1^ of (Et-4BrT)[Ni(dmit)_2_]_2_ obtained at *H* = 0, 4, 6, 8 T. The red arrow is the temperature where *χT* vs *T* plot shows minimum value. The inset is an extended region between 5 K and 15 K.

The temperature dependences of electric resistivity of (Et-4BrT)[Ni(dmit)_2_]_2_ at ambient pressure, 1.0 GPa, and 1.9 GPa are shown in Fig. S10. The insulating feature below about 50 K are detected at ambient pressure data. The resistivity gradually shows conductive feature at 1.0 GPa and 1.9 GPa. The behavior is consistent with the results reported by Kusamoto *et al.* in supplementary information in Ref.17.


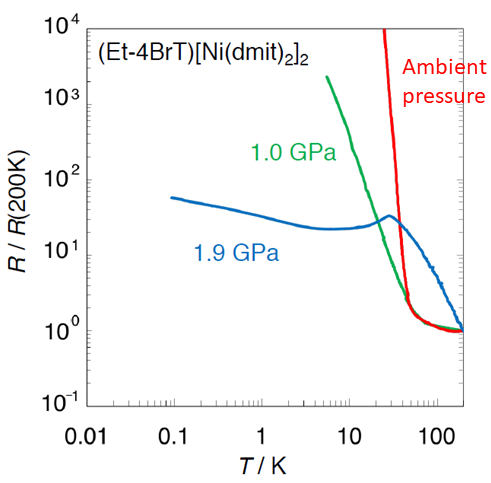


Figure S10 Temperature dependences of resistivity of (Et-4BrT)[Ni(dmit)_2_]_2_ obtained at ambient pressure, 1.0 GPa, and 1.9 GPa.

References for Supplemental Information

R1 Fukuoka, S., Horie, Y., Yamashita, S. & Nakazawa, Y. Development of heat capacity measurement system for single crystals of molecule-based compounds. *J. Therm. Anal. Calorim.* **113**, 1303–1308 (2013).

R2 Imajo, S., Yamashita, S., Fukuoka, S. & Nakazawa, Y. Construction of relaxation calorimetry for 10^1-2^ micro-gram samples and heat capacity measurements of organic complexes. *J. Therm. Anal. Calorim.* **123**, 1871-1876 (2016).

R3 Kubota, O. & Nakazawa, Y. Construction of a low-temperature thermodynamic measurement system for single crystal of molecular compounds under pressures. *Rev Sci Instrum.* 79, 053901-1-6 (2008).

R4 Nakazawa, Y., Yoshimoto, R., Fukuoka, S. & Yamashita, S. Investigation on electronic states of molecule-based compounds by high-pressure ac calorimetry. *Curr Inorg Chem.* 4, 122-134 (2014).

R5 Nakazawa Y. & Kanoda, K. Electronic structure of insulating salts of κ-(BEDT-TTF)_2_X family studied by low-temperature specific-heat measurements. *Phys. Rev.* B **53**, R8875-R8878 (1995).
